# Correction: ‘Dental cementum virtual histology of Neanderthal teeth from Krapina (Croatia, 130–120 kyr): an informed estimate of age, sex and adult stressors’ (2022), by Cerrito *et al.*

**DOI:** 10.1098/rsif.2024.0069

**Published:** 2024-02-21

**Authors:** P. Cerrito, A. Nava, D. Radovčić, D. Borić, L. Cerrito, T. Basdeo, G. Ruggiero, D. W. Frayer, A. P. Kao, L. Bondioli, L. Mancini, T. G. Bromage

**Keywords:** dental cementum, life-history evolution, fossil hominins, Neanderthals, virtual histology, synchrotron X-ray microtomography

*J. R. Soc. Interface* **19**, 20210820 (Published online 23 February 2022). (https://doi.org/10.1098/rsif.2021.0820)

It has come to our attention that some of the funding information was omitted from the Funding section of the article.

The Funding section should read: ‘The research was supported by a National Science Foundation Graduate Dissertation Research Improvement Grant (to P.C., grant no. 2018357), by the NYU MacCracken Fellowship (to P.C.), by the Marie Skłodowska-Curie Actions Individual Fellowship (to A.N., grant no. H2020-MSCA-IF-2018-842812) and by the European Union's Horizon 2020 research and innovation programme under the Marie Skłodowska-Curie grant agreement no. 846856 (to D.B.)’.

This has been corrected on the publisher's website.

